# Assessing the Accuracy of Quantitative Molecular Microbial Profiling

**DOI:** 10.3390/ijms151121476

**Published:** 2014-11-21

**Authors:** Denise M. O’Sullivan, Thomas Laver, Sasithon Temisak, Nicholas Redshaw, Kathryn A. Harris, Carole A. Foy, David J. Studholme, Jim F. Huggett

**Affiliations:** 1 Molecular Biology, LGC Ltd., Queens Road, Teddington TW11 0LY, UK; E-Mails: sasithon.temisak@lgcgroup.com (S.T.); nicholas.redshaw@lgcgroup.com (N.R.); carole.foy@lgcgroup.com (C.A.F.); jim.huggett@lgcgroup.com (J.F.H.); 2 Biosciences, University of Exeter, Geoffrey Pope Building, Stocker Road, Exeter EX4 4QD, UK; E-Mails: twl207@exeter.ac.uk (T.L.); d.j.studholme@exeter.ac.uk (D.J.S.); 3 Department of Microbiology, Virology and Infection Control, Great Ormond Street Hospital for Children NHS Trust, Great Ormond Street, London WC1N 3JH, UK; E-Mail: Kathryn.harris@gosh.nhs.uk

**Keywords:** molecular profiling, metagenomics, 16S rRNA gene, amplicon sequencing, whole genome shotgun sequencing, control material

## Abstract

The application of high-throughput sequencing in profiling microbial communities is providing an unprecedented ability to investigate microbiomes. Such studies typically apply one of two methods: amplicon sequencing using PCR to target a conserved orthologous sequence (typically the 16S ribosomal RNA gene) or whole (meta)genome sequencing (WGS). Both methods have been used to catalog the microbial taxa present in a sample and quantify their respective abundances. However, a comparison of the inherent precision or bias of the different sequencing approaches has not been performed. We previously developed a metagenomic control material (MCM) to investigate error when performing different sequencing strategies. Amplicon sequencing using four different primer strategies and two 16S rRNA regions was examined (Roche 454 Junior) and compared to WGS (Illumina HiSeq). All sequencing methods generally performed comparably and in good agreement with organism specific digital PCR (dPCR); WGS notably demonstrated very high precision. Where discrepancies between relative abundances occurred they tended to differ by less than twofold. Our findings suggest that when alternative sequencing approaches are used for microbial molecular profiling they can perform with good reproducibility, but care should be taken when comparing small differences between distinct methods. This work provides a foundation for future work comparing relative differences between samples and the impact of extraction methods. We also highlight the value of control materials when conducting microbial profiling studies to benchmark methods and set appropriate thresholds.

## 1. Introduction

Microbial molecular profiling allows the study of genetic material from a microbiome. Advances in whole genome sequencing (WGS) methods have made comprehensive genetic analysis possible, facilitating the development of the field of metagenomics. Large scale metagenomic studies have provided insight into a wide variety of diverse ecosystems, including the oceans [1], the human body [2], soil [3] and eventually all global environments [4].

Amplicon sequencing can also be used for measuring the microbiome and uses PCR to amplify conserved orthologues. The most popular target, the 16S ribosomal RNA (16S rRNA) gene, has been used in taxonomic studies for classifying bacteria at the molecular level for decades [5]. It is a key genetic marker in phylogenetic studies [6] and consequently is a popular alternative to WGS for microbiome analysis [7,8].

The abundance, as well as type, of the organisms measured is commonly reported in microbial profiling studies. As with most molecular methods a profiling experiment requires a series of distinct steps including sample collection, nucleic acid extraction, library preparation, sequencing, data processing and analysis. Each step has the potential to introduce error [9,10,11] which comprises both random and systematic variation. Random variation occurs when making repeated measurements and is the component used to determine variance and precision. Systematic errors lead to biases and potentially incorrect findings [12]. They may be inherent to various instruments and associated methodologies; importantly they can be compensated for, but only if they are known. When performing microbiome measurements each step of the experimental protocol can contribute to both types of error.

Repeat measurements allow researchers to assess random variation, but systemic variation is more difficult to evaluate and methods for evaluating microbiomes arguably present a particular challenge as the quantification of multiple different sequences are performed. This leads to the added complication of assigning the sequences to a given organism. This will inevitably lead to challenges with data reproducibility, a fact that is further augmented by the different protocols and instruments as well as the vast range of tools available for metagenomic data analysis. Certain guidance which has outlined the requirements for publishing metagenomic data has aimed to standardise and improve the quality of the reporting in the literature [13].

Biases can be investigated using control materials [11,14,15,16] to interrogate the different software packages, the impact of sample preparation, 16S rRNA primer choice and amplicon preparation, direct sampling and library preparation. In addition to this, the performance characteristics of the different sequencing platforms can be investigated. We have previously described the application of a control material to compare different library preparation methods [15]. In this study we build on this work by investigating the impact associated with 16S rRNA assay choice and design, and compare amplicon sequencing approaches to WGS. We used different informatics strategies to further evaluate the data to assess the impact of data handling on results. To add rigour to our findings we further evaluated the control material using a series of additional non-sequencing methods including digital PCR (dPCR). dPCR can precisely quantify nucleic acids by performing a limiting dilution of your sample so that there are either no or one target molecule in a very large number of individual reactions. The number of target molecules is counted in a digital format [17,18]. This study uses a well characterised control material to assess multiple important factors in a microbial profiling study, providing a measure of their precision and bias.

## 2. Results and Discussion

### 2.1. Initial Analysis and Characterization of the Metagenomic Control Material (MCM)

Previous studies have exploited control materials to compare metagenomic approaches [11,14,15,16]. Typically, control materials have been initially quantified using either fluorescence or qPCR and then used to interrogate different sequencing methods. If the findings of the different methods disagree it can be assumed that there must be a bias (systematic error) but it can be difficult to conclude which if either method is correct. Furthermore, when they agree it might be incorrect to assume that this reflects accuracy as the two methods may both be biased. In this study we prepared a control material and used two independent methods (direct fluorescence and dPCR) to initially characterise the material thus highlighting potential sources of bias prior to assessing a number of different molecular microbial profiling methods using high throughput sequencing.

**Figure 1:**
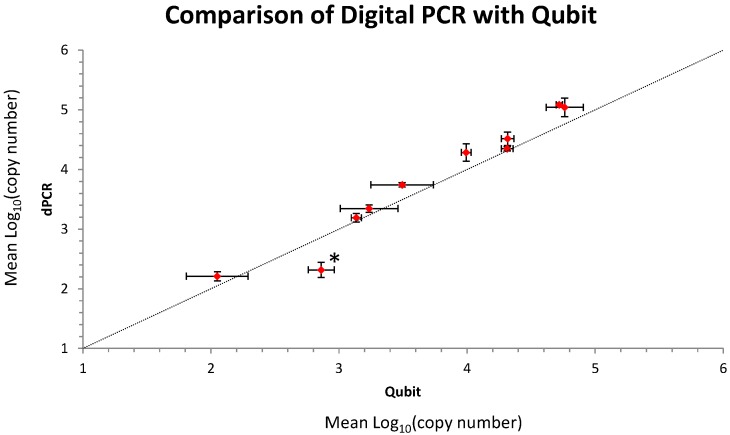
*X*/*Y* plot comparing the mean log_10_ (copy number) of each of the bacterial gDNA’s as estimated by the Qubit fluorometer and digital PCR (dPCR). The asterix indicate significance using *t*-test and Bonferroni correction. Error bars indicate 95% confidence intervals. The slope is significantly different than zero (*p* value < 0.000002) but not significantly different from 1 (*p* value: 0.16).

The composition of the metagenomic control material (MCM) containing 10 different pathogenic bacterial species (5 Gram negatives: *Neisseria meningitidis*, *Klebsiella pnuemoniae*, *Escherichia coli*, *Pseudomonas aeruginosa*, *Acinetobacter baumanii* and 5 Gram positives: *Streptococcus pneumoniae*, *Staphylococcus aureus*, *Streptococcus pyogenes*, *Streptococcus agalactiae* and *Enterococcus faecalis*) was as previously described (Table S1) [15]. The MCM was prepared using triplicate estimations of the quantity of the respective bacterial genomic DNAs (gDNAs) using the Qubit BR (broad range) dsDNA (double-stranded DNA) assay (Life Technologies, Carlsbad, CA, USA) and performing measurements of the fluorescence as determined by the Qubit 2.0 Fluorometer (Life Technologies). The general agreement between the initial fluorometric quantification of the individual gDNAs and subsequent quantification of the respective gDNAs in the MCM using dPCR was good (Figure 1). Further analysis of the respective data sets showed that there was generally either no significant difference between the respective estimations or a slight increase in the dPCR value with the exception of *P. aeruginosa* which showed a significant ~3-fold decrease in the dPCR estimation when compared to fluorescence (Figure 1). The MCM was shown to be stable for the duration of the study (Figure S1).

Quantification of DNA by direct fluorescence and dPCR offers two completely independent methods for estimating gDNA copy number. Fluorescence methods must be compared to a calibration curve; in the case of the Qubit assay, Lambda DNA is quantified using absorbance at 260 nm (A_260_). dPCR offers an absolute estimation of a small region of the extracted gDNA that is independent of a standard curve. When two completely independent methods agree this increases confidence in the findings, this is important for the value of any quality control material used in the evaluation of precision and bias of other methods. Where the dPCR over-estimates the value it is <2.5-fold which depending on the level of precision required could impact on findings. This is likely to be due to either direct fluorescence underestimation or to dPCR overestimation. Fluorescence underestimation could be caused if the calibrator is unsuitable or if it is initially quantified inaccurately. dPCR overestimation of a well-designed and optimised assay can only occur due to single stranded partitioning of DNA [19]. The reason for the differences in the measurement of *P. aeruginosa* was not clear but it was not due to auto-fluorescence leading to overestimation by the Qubit (Table S2) nor DNA fragmentation leading to underestimation by dPCR (Figure S2). However, despite the fact that this template did not follow the trend of the other organisms (Figure 1), the difference between the estimation by dPCR and Qubit was small (~3-fold) so the MCM was used to interrogate different sequencing methods.

### 2.2. Molecular Profiling Using Different Sequencing Strategies

**Figure 2:**
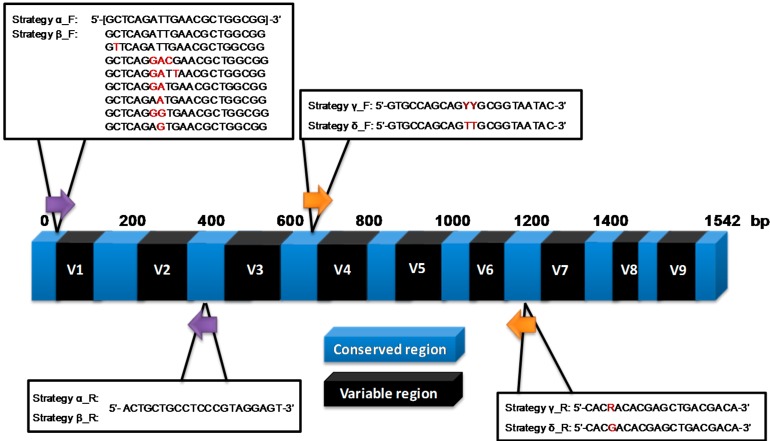
A summary of the four different primer strategies for amplification of variable regions 1, 2, 4, 5 and 6 of the 16S rRNA gene (positioning is based on the *E. coli* 16S rRNA gene). Highlighted in red are differences in base sequence.

We initially used the MCM to investigate amplicon sequencing by four different primer strategies targeting two different variable regions of the 16S rRNA gene in order to evaluate their impact on quantification (Figure 2). Two strategies (α and β) targeted an amplicon that spanned variable regions 1 and 2 and to compare the measurements using different variable regions we targeted an amplicon to span variable regions 4, 5 and 6 (strategy γ and δ). Both combinations of regions have been investigated previously [20,21,22]. However we redesigned the primers to the respective conserved regions to investigate the impact of different primer strategies on the results. Each experiment performed three replicate runs of the whole protocol including 16S rRNA PCR.

Strategy α was designed to highlight how a single primer which perfectly matched the conserved regions spanning variable regions 1 and 2 of Gram-negative but not Gram-positive bacteria would perform on quantification. This was compared to using mixes of different specific primers to the same priming site, (strategy β). To target the conserved regions spanning variable regions 4, 5 and 6, we used the popular degenerate base method (strategy γ), to understand its impact on quantification, and a novel strategy that uses a single primer pair but capitalises on nucleotide cross priming [23] (strategy δ) (further description of the approaches is in Section 3.3).

**Figure 3:**
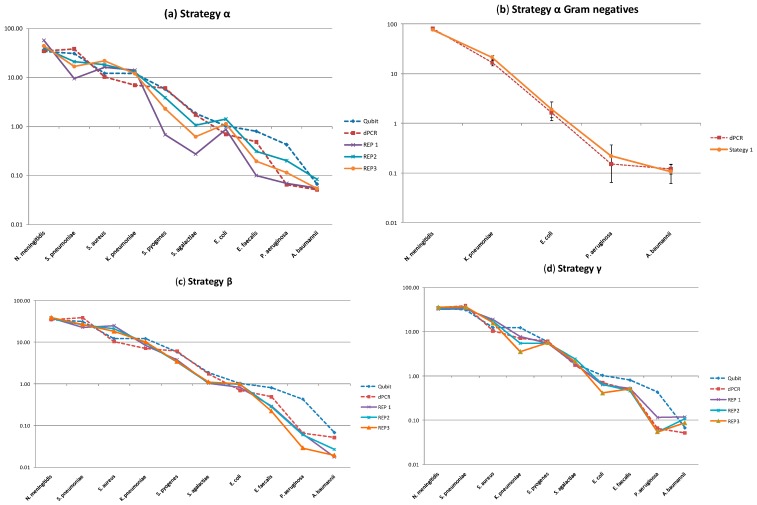

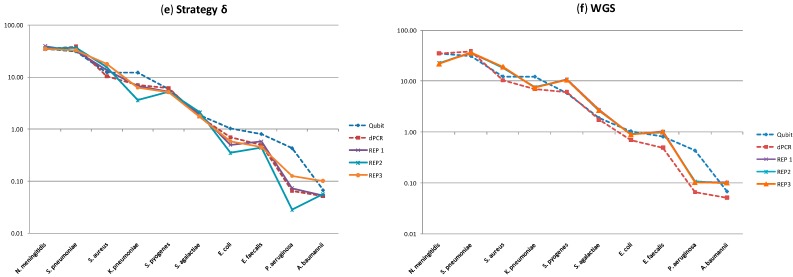
Relative copy number, expressed as a percentage of the metagenomic control material, from amplicon sequencing using different strategies amplifying the 16S rRNA. The error bars refer to the 95% confidence interval. Dashed blue line with a triangle represents the Qubit value, the dark red dashed line with a box represents the dPCR value, the purple line with cross is replicate 1, blue line with asterix replicate 2 and orange line with triangle replicate 3 of the sequencing approaches. (**a**) The α strategy which targets the Gram-negative members of the MCM; (**b**) expanding the results for the Gram-negative species and (**c**) the β strategy using multiple forward primers with a single reverse primer to target 16S rRNA variable regions 1 and 2; (**d**) The γ strategy using a degenerate primer and (**e**) δ strategy using the ability of T to bind to G and *vice versa* targeting 16S rRNA variable regions 4, 5 and 6; (**f**) Expresses relative composition of the MCM as determined by whole genome sequencing.

Using our standard bioinformatics approach for the amplicon sequencing of assigning species based on aligning against a database of the known MCM species 16S rRNA sequences, we noted two factors when strategy α was employed. Firstly there was considerably more inter-run variation (Figure 3a and as demonstrated by coefficient of variation in Table 1), although this appeared to be predominantly due to the increased error associated with the Gram-positive bacteria to which the primers were not specific. Secondly as the primers for strategy α bound perfectly to the conserved regions of the Gram-negative bacteria we were able to assess if differences in the variable regions (Figure S3) would lead to bias in a primer independent manner. Figure 3b demonstrated good agreement with dPCR suggesting the sequence differences present in the respective variable regions (Figure S3) did not lead to considerable bias. When a mixture of specific primers were used (strategy β) the precision was considerably improved (Table 1) and there was better agreement with the dPCR (Figure 3c). When a different variable region was investigated using strategies γ and δ we noted that there was little difference with the respective methods both being more precise than strategy α (Figure 3d,e). An earlier investigation into sources of PCR bias demonstrated choice of primers had a major influence [24], we demonstrate here that different variations of the same priming sites can also have the same effect.

To perform a similar assessment using WGS molecular profiling we performed three replicated runs of the Nextera XT library preparation method and sequenced this in multiplex on the Illumina HiSeq 2000. The Nextera protocol was chosen as it uses a low starting concentration of template (1 ng) to prepare libraries. When analysed using our standard bioinformatics approach of aligning the reads against a reference made up of the MCM species, the WGS was consistently more precise than the amplicon sequencing (Figure 3f and Table 1).

**Table 1:** The % coefficient of varation (CV) of the 5 different strategies in targeting the 10 species present in the metagenomic control material (MCM), in order of abundance high to low for Gram negative and Gram positive bacteria.

Gram	Species	Strategies (% CV)				
		α	β	γ	δ	WGS
Negative	*N. meningitidis*	19	2	5	6	1
	*K. pnuemoniae*	8	10	37	31	2
	*E. coli*	24	10	23	25	1
	*P. aeruginosa*	52	37	47	64	2
	*A. baumannii*	26	23	15	38	2
Positive	*S. pnuemoniae*	37	10	5	7	1
	*S. aureus*	15	16	9	13	3
	*S. pyogenes*	70	7	2	1	1
	*S. agalactiae*	61	3	13	10	2
	*E. faecalis*	52	14	7	16	2
Average		36	13	16	21	2

One of our key findings is that due to the high precision afforded by most of the methods, significant differences between methods are frequently observed. However this is seldom greater than two fold suggesting that researchers applying these methods should treat significant differences that are less than threefold with caution and therefore apply control materials (Table S3). Of note when comparing all the sequencing methods agreed with the dPCR measurement of *P. aeruginosa* and not the fluorescence measurement lending support to the notion that it is the fluorescence measurement that was overestimating the DNA concentration that caused this initial disagreement.

One factor that might influence precision of WGS-based measurement is the depth of coverage of a genome by WGS sequence data. For example, it might be expected that at very shallow coverage, stochastic effects might lead to high levels of imprecision that are eliminated as coverage reaches saturation. Therefore we used a bootstrapping approach to investigate the effect of coverage depth on precision.

Our results show that for sample sizes of down to 2500 reads mean results remain in line with those for the full dataset (Table S4). The smaller the sample size the larger the variation between individual runs, however the variation is still much less than that for the amplicon sequencing. Conducting this bootstrapping approach allows us to be confident that the superior precision shown by the WGS datasets when compared to amplicon approaches is not simply an artifact of coverage depth. This helps guide minimum sample size needed for future studies. However one would need to take into account the relative abundance of the organisms being targeted, the desired confidence for performing rare species calls and the fact that the database of just the species which are included in the MCM is not a full database where the results will be likely to be complicated by the associated complexity. Table S5a demonstrates the percentage of reads removed by filtering for each of the methods which showed more variability in the percentage of reads removed for amplicon sequencing when compared to WGS perhaps indicating variability in the quality of the amplicon sequencing runs. However, as we followed a strict filtering regime it should equalise the quality of the datasets so that only those reads of high quality are included in the analysis.

### 2.3. Evaluation of the Effect of Database Composition

One of the key advantages to applying synthetically made control material is that results can be compared to the organisms that are known to be present; we exploited this in our standard bioinformatics approach by restricting reference databases to MCM species only. Real applications of molecular microbial profiling do not have the luxury of *a priori* knowledge of the species present in their sample. To test the effect on the results we re-analysed our data against databases appropriate for a typical bacterial profiling study.

The amplicon datasets were reprocessed using the SILVA database, an online quality checked resource of aligned gene sequences of ribosomal RNA genes, as a reference [25] (Table S5b). Despite requiring a minimum support of 5 reads for a taxon to be called, there were still assignments to some genera that are not present in the MCM. This shows that when conducting microbial profiling studies the detection of rare species cannot always be trusted, as has previously been suggested [26]. This raises an important point, that when conducting microbial profiling studies, specific hits to databases should be viewed with a healthy skepticism and important taxonomic assignments should be verified, at least bioinformatically. For example the taxonomic identities could be verified by phylogenetic categorisation, an approach espoused by pipelines such as Phyloassigner [27]. Comparing the results to our standard approach, when using the SILVA database we consistently fail to get genus level assignments for *Klebsiella*, *Escherichia* and *Pseudomonas* across all amplicon datasets. This is particularly striking for *Klebsiella* as it is one of the more abundant species in the MCM. Failure to assign reads to these missing genera could be due to 16S rRNA conservation, read error or errors in the SILVA database. We feel the latter is a more likely explanation. We have detected errors in the SILVA database affecting those missing genera (Table S6 and Figure S4). This raises an important point, that when conducting microbial profiling studies, specific hits to databases should be viewed with a healthy skepticism and important taxonomic assignments should be verified, at least bioinformatically.

We analysed the WGS data against a reference of all completed bacterial genomes (down-loaded 25 February 2014 from ftp://ftp.ncbi.nlm.nih.gov/genomes/Bacteria/). The results show a high level of agreement, in terms of proportions of the target species, with those results generated from using our standard approach. Less than 2% of reads hit species that are not present in the MCM. However, those 2% of reads hit a very wide range of species at very low coverage; one WGS replicate had hits to all but 4 out of 1400 species aligned against. This shows the importance of setting a sensible significance threshold for declaring the presence of a species, which also need to be set in accordance with coverage depth. We can conclude from this that WGS studies can achieve accurate quantifications of the proportions of species present in a sample. However they may overestimate diversity and falsely suggest the presence of rare species.

The conclusions from both sequencing methods can only be verified by the use of such control materials which due to prior knowledge are able to test the performance of both laboratory and informatics processes. We have demonstrated by applying control materials the clear limitations regarding the level of operational taxonomic unit that can be resolved and potential problems of mis-calling organisms that are not present. Verification of findings is therefore advisable, at least by using alternative informatics methods but ideally using non sequencing based methods like PCR. The approach here could also be used to evaluate sources of specific sequencer error using either 16S rRNA amplicon sequencing or WGS approaches. Additionally further work should also include the investigation into the impact of other genomic variables such as variable GC content, epigenetics and plasmid content on sequencing as well as the impact of steps like extraction using whole cells. This study focused on single strains representing each organism but strain variation should also be investigated in future control panels.

## 3. Experimental Section

### 3.1. Preparation of Metagenomic Control Material

Microbial gDNA was sourced from ATCC (LGC Standards, Teddington, Middlesex, UK) from 10 organisms with a 33%–67% GC content, 1.85–6.26 MB genome size and a range of 16S rRNA copy number (4–8) (Table S1) and mixed to prepare a metagenomic control material (MCM). The quantitative composition of the panel was prepared to span over two orders of magnitude ranging from ~100 (*A. baumannii*) to >50,000 (*N. meningitidis*) genomic copies per µL. The MCM was prepared as previously described [15]. The concentration of the respective gDNAs (ng/µL) were determined using the Qubit dsDNA BR Assay Kit on the Qubit Fluorometer. Three replicate measurements were observed and the mean value was reported. The concentrated material (25 ng/µL) was incubated at 4 °C for 3 h on a tube rotator. This material was diluted in TE pH 7.0 buffer to a working stock concentration of 1 ng/µL.

Stability of the MCM stored at −20 and −80 °C was determined at 0, 7, 14, 90, 180 and 360 days using qPCR assays *ctrA* targeting *N. meningitidis* and *ply* targeting *S. pneumoniae* (Table S7). The results from the long term stability of the MCM stored at −80 °C at the 3, 6 and 12 month time points is reported in Figure S1. Reactions were performed in triplicate, including a no template control, in 1× Fast Probe Master Mix with ROX (Biotium, Hayward, CA, USA), 900 nM of each primer, 200 nM of the hydrolysis probe in nuclease free water (Ambion, Foster City, CA, USA) in a total reaction volume of 20 µL. Thermocycling conditions were 95 °C 10 min followed by 45 cycles of 95 °C for 15 s and extension at 60 °C for 1 min. The data was analysed using automatic settings on ABI 7900HT Sequence detection systems version 2.4.1 SDS2 software (Applied Biosystems, Foster City, CA, USA)

### 3.2. Microfluidic Digital PCR

For the accurate quantification of the members of the MCM microfluidic dPCR was performed. Each 4 µL reaction contained: 1× TaqMan Gene Expression Master Mix (Applied Biosystems), 1× GE sample loading reagent (Fluidigm, San Francisco, CA, USA), specific primers covering each constituent of the MCM and 1.2 µL of MCM. The templates were diluted to the appropriate concentration to be detected by the dPCR, nuclease free water was included as a no template control (NTC). The reactions were performed on a 37k IFC for quantitative dPCR chip (Fluidigm) containing 770 × 0.84 nL partitions in each of the 48 panels. Reactions were performed in triplicate using the following conditions 95 °C 10 min followed by 45 cycles of 95 °C for 15 s and 60 °C for 1 min. The dPCR results were analysed using the Digital PCR Analysis Software version 4.0.1 (Fluidigm). The software counted the number of positive chambers and makes a correction by converting from positive compartments to number of target molecules using Poisson statistics, to account for the fact that a chamber may contain more than one target molecule DNA. As our specific target gene can be found on a single location on bacterial chromosome, the absolute count of target molecule was assumed equal to the absolute count of bacterial genomic copy number. The absolute gDNA copy number of each bacterium in the MCM was presented with 95% confidence intervals. The experiments were performed in triplicate digital chip running independence. The dMIQE (Minimum Information for publication of Quantitative Digital PCR Experiments) checklist for this study can be found in Table S8 [28]. Figure S5 shows examples of positive and negative amplification from the dPCR.

### 3.3. Amplicon Sequencing

#### 3.3.1. PCR

There were four different strategies employed for amplification of the 16S rRNA gene (Figure 2). Strategy α was designed to be specific for the Gram-negative bacteria in the MCM using a single forward and reverse primer to flank variable regions 1 and 2 with no degenerate bases. Strategy β targeted variable regions 1 and 2 employing a combination of forward primers and one single reverse primer to provide specificity to all MCM species. Strategy β used degenerate bases in the forward and reverse primers to target variable regions 4–6. The primers used in strategy δ for amplifying variable regions 4-6 take advantage of the fact that T binds not only to A but can also bind to a lesser extent to G [23] to generate a single forward primer with a single reverse primer.

Experimental conditions were as recommended by the manufacturer (Roche 2012). Optimal primer concentrations and annealing temperatures were determined prior to preparing the amplicon for sequencing. In brief, 1.25 units of FastStart High Fidelity DNA polymerase (Roche Applied Science, Mannheim, Germany), 1× Fast Start buffer (Roche), 200 µM dNTPs (Roche), 900 nM of each primer for variable regions 4–6 and 400 nM of each primer for variable regions 1 and 2, were added to 1 ng of MCM in a background of 50 ng human gDNA (Promega, Madison, WI, USA) as the template and made up to a final volume of 25 µL in nuclease free water (Ambion). The reactions were performed on a Gene Amp PCR system 9700 (Applied Biosystems) using the following conditions; 94 °C 3 min, 35 cycles of 94 °C 15 s, 61 °C 45 s and 72 °C 60 s and an extension step 72 °C for 8 min. Amplicons (assay 1 and 2, 337 bp size and assay 4, 5 and 6, 564 bp size) were visualised using the Agilent DNA 1000 chip and kit on the 2100 Bioanalyser (Agilent Technologies, Salt Lake City, UT, USA) to confirm the expected amplicon size and purity. Cleanup of the PCR reactions were performed using QIAquick PCR purification kit (Qiagen, Alameda, CA, USA), following manufacturer’s instructions. The PCR products were eluted with 50 µL 1× TE buffer (Roche). PCR products were visualised using the Agilent Bioanalyzer 2100 to verify product size and quantified using the Qubit 2.0 Fluorometer with Qubit dsDNA BR Assay Kit.

#### 3.3.2. Amplicon Sequencing

Three replicate sequencing experiments were performed according to the manufacturer’s instructions (standard protocols for Roche GS Junior 454 Sequencing using the Lib-L method March 2012 version). This approach was previously found to be preferable compared to the Lib-A method [15]. Adapter ligation was performed according to the Lib-L library preparation method with 500 ng PCR product. Libraries were then purified using Agencourt AMPure beads (Beckman Coulter, Beverly, MA, USA) (bead to DNA ratio of 1.6:1), visualised using the BioAnalyser 2100 to determine ligation efficiency and quantified using the Qubit 2.0 Fluorometer with Qubit dsDNA BR Assay Kit to estimate DNA copy number. Amplicons were pooled in equimolar concentrations prior to emulsion PCR which was performed with a Lib-L emulsion PCR kit (Roche) using 2 × 10^7^ DNA molecules with 1 × 10^7^ beads (2 to 1 ratio). DNA sequencing was performed using the Roche GS Junior Titanium sequencing kit and PicoTiterPlate. Data was processed using the Roche Shotgun sequencing pipeline.

#### 3.3.3. Data Analysis

The sequence data was filtered based on the steps taken in the high stringency pipeline of the Human Microbiome Project [29]. The sequence of the PCR primers was used to group the sequence reads for each target amplicon, allowing 2 base pair mismatches to the PCR primer. Chimeras from the PCR were eliminated using ChimeraSlayer [16]. Sequences were trimmed when the average quality within a 50 bp sliding window fell below 35. Reads were removed if the length was 10% greater than the expected amplicon length or if after quality trimming, they were shorter than 200 bases. Reads containing ambiguous base calls or homopolymer runs longer than 8 nucleotides were removed.

For our standard analysis approach, the filtered reads were given a taxonomic assignment by performing a megaBLAST search (a module of BLAST) [30] against a custom database of the 16S rRNA sequences of the species contained within the LGC MCM. Using a database containing only the target organisms allows a more accurate quantification of the sequencing results and is thus superior for assessing the reproducibility and precision of the sequencing. This pipeline is not designed to evaluate the informatics analysis but to provide the most accurate data for evaluating the other steps in the process. The BLAST files were processed with MEGAN (MEtaGenome Analyser) [31], using default settings with the exception of restricting BLAST hits to those within 1% of the score of the top hit. MEGAN calculates a taxonomic classification for each reads based on the lowest common ancestor of those qualifying blast hits. Those reads that receive species level assignments are then normalised by the species’ 16S rRNA copy number and used to calculate the relative abundances of each species.

To assess the effect of choice of reference database the amplicon reads were also analysed by performing a megaBLAST search [30] against the SILVA database [25]. The resulting taxonomic assignments were processed with MEGAN [31] using default settings. Relative abundances were calculated based on genus level assignments. The checklist for the MIxS (minimum information about any (x) sequence) is in Table S9 [13].

### 3.4. Whole Genome Sequencing

Whole genome sequencing was performed by LGC Genomics GmbH (Berlin, Germany). Two × 100 bp paired end libraries were prepared using 25 ng of DNA from 3 individual aliquots of the MCM with the Nextera XT kit (Illumina, San Diego, CA, USA). Sequencing was performed on the HiSeq 2000 (Illumina).

#### Data Analysis

The whole genome sequence data was quality checked using Fast-QC [32]; this analysis suggested the presence of adaptor contamination. The NGS QC Toolkit [33] and Fastq-mcf [34] were used to quality filter the data. This included removal of the indicated adaptor contamination, read trimming and exclusion of lower quality reads. After low quality bases were trimmed from the ends of reads, those with less than 30 bases remaining were removed. Reads with less than 90% of bases of quality 30 or greater were filtered out. Any read where the corresponding paired read was removed was placed in an unpaired file which was then treated separately in the analysis.

In our standard bioinformatics approach, the reads were aligned to a custom database of the genome sequences from the species in the MCM using Bowtie 2 [35], using default settings and taking the paired and unpaired read files as input. The alignment file was then processed using SAMtools [36] to generate read counts for each species, which were then normalised by genome size to give relative abundances for each species. Hits to plasmids were excluded as their copy number is unknown.

When re-analysing the WGS data to assess the effect of the choice of reference database, the NCBI completed bacterial genomes (downloaded 25 February 2014 from ftp://ftp.ncbi.nlm.nih.gov/genomes/Bacteria/) was used to taxonomically bin the filtered WGS reads. The alignment was then processed in the same way as in the standard approach.

To assess the effect of sample size on the precision of the WGS results we conducted a bootstrapping study. 1000 subsamples of each of the filtered WGS read sets were generated (with replacement) for subsample sizes of 500, 1000, 2500, 5000, 10,000 and 30,000 (Table S4). Reads were sampled from the two paired files and the unpaired reads in proportions corresponding to the dataset. These subsamples were then analysed following our standard approach.

The checklist for the MIxS (minimum information about any (x) sequence), as mentioned for the amplicon sequencing results, is in Table S9.

## 4. Conclusions

A well characterised control material enables researchers to interrogate the performance of their community profiling methods. In this study we have investigated the performance of amplicon sequencing (using different priming strategies) and WGS using our metagenomic control material. We found that the sequencing methods had high precision, with the WGS sequencing results having the highest intermediate precision. While agreement between methods was generally good, significant differences were observed; however this was due to the high precision of the methods and was rarely greater than twofold. When the sequencing methods were compared with frequently used bioinformatics pipelines, organisms were missed or incorrectly identified. The type of control materials described and applied in this study provide a valuable tool for validating the library preparation, sequencing and informatics stages of a microbial profiling experiment. Furthermore methods used to perform the comparisons outlined by this study would enable new laboratories embarking on molecular microbial profiling experiments to select their procedures and provide a simple method for established laboratories to compare findings.

## Supplementary Materials

Supplementary materials can be found at http://www.mdpi.com/1422-0067/15/11/21476/s1.
